# From probe to prodrug: immunoproteasome activity as a trigger for disease-selective therapeutics

**DOI:** 10.1038/s41392-026-02835-w

**Published:** 2026-08-12

**Authors:** Cody A. Loy, Yijun Gu, Samuel C. Kim, Mariam V. Mohagheghi, Noah B. Trask, Marina Suarez-Pizarro, Lisa E. Wagar, Claudia A. Benavente, Darci J. Trader

**Affiliations:** 1 Department of Pharmaceutical Sciences, University of California, Irvine, CA USA; 2 Robert A. Mah Molecular Innovation Center, University of California, Irvine, CA USA; 3 Department of Physiology and Biophysics, University of California, Irvine, CA USA; 4 Adeline Yen Mah Vaccine Center, University of California, Irvine, CA USA; 5 Institute for Immunology, University of California, Irvine, CA USA; 6 Center for Virus Research, University of California, Irvine, CA USA; 7 Department of Developmental and Cell Biology, University of California, Irvine, CA USA; 8 Chao Family Comprehensive Cancer Center, University of California, Irvine, CA USA; 9 Deparment of Chemistry, University of California, Irvine, CA USA

**Keywords:** Target validation, Target validation

## Abstract

Developing novel strategies that can exploit a cell's endogenous machinery to selectively release toxic compounds in diseased cells, while leaving healthy cells unaffected, is a challenging task to accomplish. An established approach involves antigen recognition on tumor cells, enabling targeted delivery of prodrugs in which a cytotoxic compound is appended to an antibody by a cleavable linker. However, this strategy depends on the presence of highly expressed surface markers for which antibodies can be developed, limiting its applicability to cancers that display these markers. A new approach that could rely on disease-specific expression of an intracellular enzyme, which can be more broadly targeted, could expand the therapeutic potential of prodrugs clinically. Here, we demonstrate that the immunoproteasome, an isoform of the standard proteasome that is upregulated under conditions of inflammation, has the potential to be harnessed as a prodrug release enzyme. We conjugate the highly toxic and widely used therapeutic cargo MMAE onto an immunoproteasome-selective peptide and demonstrate its selective release in cancerous cells at low nM to pM concentrations, while healthy cells remain viable. We also establish the first translational validation that using immunoproteasome prodrugs in vivo leads to a significant reduction in tumor volume without significant toxicities in small cell lung cancer. We anticipate this approach to be broadly applicable, as the immunoproteasome is upregulated in a variety of cancers and does not require antibody recognition to be effective.

## Introduction

A major challenge in drug discovery remains the ability to target a diseased cell population while leaving the healthy tissue unaffected. Several strategies have been applied to overcome this such as targeting a protein that is overexpressed in the diseased cell population, in silico prediction and optimization,^1,2^ and tissue-specific delivery systems.^3^ Although these methods have shown promise in improving off-target toxicities, identifying methods that have wide therapeutic windows is still a challenge. One method that has been employed widely is the use of masking a toxic compound with a pro-moiety sequence that can be removed once the compound has entered the diseased environment.^4^ This removal can be performed by external sources such as thermal- or photo-cleavable groups, enzymes that are upregulated or only expressed in the diseased environment, or by metabolic changes to the molecule that can be converted into the active compound upon entering the diseased cell.^5,6^ One of the most popular approaches to this prodrug strategy is the use of antibody-drug-conjugates (ADCs).^7,8^ ADCs typically rely on the recognition of an antigen expressed on the surface of the diseased cell that can direct the therapy to, leading to internalization of the compound into lysosomes, where the acidic environment cleaves the pro-moiety sequence, allowing the toxic compound to be released and perform its intended mechanism of action. Although this method is effective at treating tumors with known antigens that can allow for the development of selective ADCs, other tumors that are more evasive remain a challenge to treat using this method.^9^ An approach that still relies on caging the compound from eliciting toxicity to healthy cells but can be liberated by an enzyme that is only being expressed in the cancerous cells, could alleviate the need for antibody recognition in these more difficult to treat cancers.

**Fig. 1:**
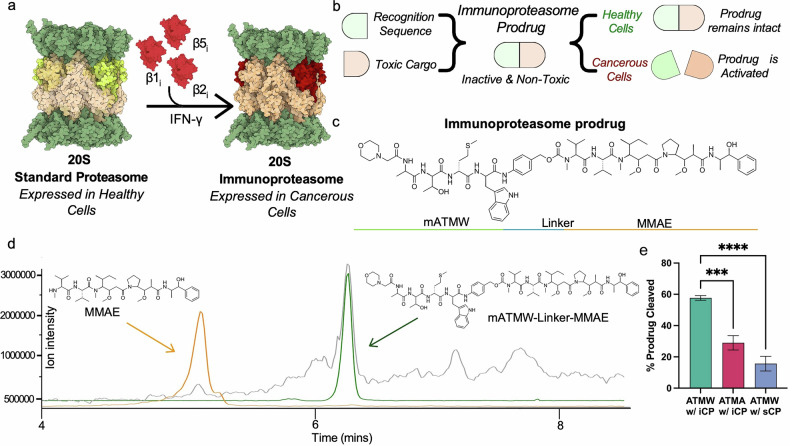
Immunoproteasome-mediated release of cargo. **a** Under conditions of inflammation the 20S immunoproteasome is synthesized by incorporating altered catalytic subunits β1i, β2i, or β5i. **b** Schematic of proposed immunoproteasome prodrug mechanism. Caging a cytotoxic compound with an immunoproteasome recognition sequence will render the drug inactive in healthy cells that do not express immunoproteasome. In cancerous cells that have an elevated expression and increased activity of immunoproteasome, the prodrug will be cleaved and cytotoxic cargo released. **c** Structure of proposed prodrug (morpholine-Ala-Thr-Met-Trp-linker-MMAE). ATMW is recognized by the iCP through interactions within the P1-P4 specificity pockets. Upon recognition, hydrolysis occurs after the tryptophan, liberating the linker-MMAE only in diseased cells. The self-immolative linker (PABC) self-disassembles to release the active MMAE to perform its intended cytotoxic function. **d** LCMS trace of prodrug incubated with immunoproteasome after 1 h. Total ion chromatogram (gray), Intact prodrug (green), and free MMAE (orange) could be detected. **e** Percent prodrug release after 1 h of incubation of ATMW prodrug with iCP (teal), or sCP (blue), and control prodrug ATMA with iCP (maroon)

The proteasome is a multi-catalytic protease complex that is responsible for the majority of protein degradation in eukaryotic cells.^10^ It is comprised of 4 sets of heptoheteromeric rings, 2 of which house the *α*-subunits that act as gates into the 2 sets of β-subunits.^11^ Within the β-subunits, 3 are catalytically active (β1, β2, and β5) and are responsible for the cleavage of proteins into small peptide fragments. In addition to the standard proteasome (sCP) that is expressed across all cell types, another isoform of the proteasome is only expressed upon exposure to inflammatory cytokines such as interferon gamma (iFN-γ). The immunoproteasome (iCP) is structurally homologous to the sCP; however, during its assembly, altered catalytic subunits are incorporated that give the iCP distinct cleavage preferences (Fig. 1a). The new catalytic subunits (β1i, β2i, and β5i) accept the same protein substrates, yet the cleaved peptides are altered to produce more major histocompatibility complex-I (MHCI) compatible peptides.^12–14^ Targeting the sCP and iCP by inhibition of its *N*-terminal Threonine (Thr1) has proven to be an effective strategy at treating cancers as well as autoimmune disorders.^15–18^ Inhibition of the iCP has become a more promising strategy, as its selective expression in diseased cell populations can improve off-target effects seen with most sCP inhibitors.^19,20^ To gain selectivity for the iCP over the sCP, interactions within the catalytic subunits of the unprimed substrate channel can be taken advantage of when developing inhibitors.^21^ Although the inhibition strategy has proven to be effective in some cases, the sCP and iCP’s therapeutic potential has yet to be fully harnessed.

Previously, we were interested in developing an activity-based probe that would allow for monitoring iCP activity in cells. To do this, a one-bead-one-compound (OBOC) library was developed and screened against the iCP to identify the most selective recognition sequence. From this screen, the 4-mer peptide Ala-Thr-Met-Trp (ATMW) was identified to be selectively cleaved by the iCP over the sCP.^22^ This recognition sequence has since been used to develop an activity-based fluorescent probe and luminescent probe.^22–24^ Recently, this peptide has been shown to be effective in harnessing the iCP as a prodrug-releasing enzyme where ATMW was appended to Doxorubicin and ARV-771, leading to toxicity or protein degradation in diseased cells, respectively, while healthy cells remained viable.^25^ This was the first demonstration of harnessing an isoform of the proteasome as an effective prodrug uncaging enzyme. Although this expands what is possible with targeting the iCP for therapeutics, we wished to push the limits of this technology and see if this is an approach capable of translating results in vivo.

The present work addresses the central unanswered question of whether iCP-mediated prodrug activation can enable safe and effective delivery of a clinically validated but systemically intolerable payload, and whether this strategy can succeed in vivo. To this end, we developed an immunoproteasome prodrug containing MMAE that directly challenges the system with a highly potent auristatin that typically requires monoclonal antibody-based delivery and is associated with severe off-target toxicity when administered freely. We show that our prodrug exhibits robust, sub-nM to pM potency selectively in cancerous cells expressing iCP, while remaining inert in healthy cells expressing mostly sCP, establishing functional discrimination that is substantially stronger than observed with prior payloads. Mechanistic studies further demonstrate that iCP mediated release of MMAE is required for toxicity. Most notably, we show that systemic administration of the prodrug achieves durable tumor control in vivo without the toxicity observed with free MMAE, thereby demonstrating that iCP release can generate a therapeutic window comparable to clinically used targeted delivery systems, but without the reliance on antibody targeting, receptor expression, or antibody trafficking. In addition, we achieved significant tumor reduction and remission with as low as 0.8 mg/kg, significantly lower than what is seen with typical FDA-approved ADCs of MMAE.^26^ Collectively, these results advance the ATMW platform from a chemical tool to a generalizable, mechanistically understood, and translationally validated prodrug strategy, capable of addressing a class of therapeutics that have historically required complex biologic delivery systems.

## Results

### Developing an immunoproteasome prodrug strategy

For a prodrug strategy to be effective, there are several factors that need to be considered and validated before it can be designated a successful approach. One of these is the establishment of a source of uncaging that is only expressed in a diseased cell population (Fig. 1b). The immunoproteasome is an isoform of the proteasome that is expressed upon exposure to inflammatory cytokines. This has made the iCP an attractive target for several diseases, including cancer. To establish models for our system, we identified cell lines with varying levels of iCP, including cells with high endogenous expression, cells with inducible expression, and cells with little to no iCP. In previous studies, we have shown that several blood cancers exhibit high levels of iCP endogenously, with Ramos B-cell lymphoma displaying particularly high iCP activity and would serve as an excellent positive control (Supplementary Fig. S1).^23,25^ For our fluorescent probe development, we used A549 non-small cell lung cancer cells, which demonstrated inducible β5i expression in response to IFN-γ exposure.^27–29^ MRC-5 lung fibroblasts and HEK-293T cells were selected as negative controls, as they both have low β5i expression, although MRC-5 fibroblasts can upregulate β5i upon IFN-γ treatment (Supplementary Fig. S1).

After establishing model cell lines to validate our prodrug mechanism, our next step was to synthesize the prodrug and several corresponding controls. The synthesis of morph-ATMW had been previously developed for our Doxorubicin prodrug.^25^ To remain consistent with several FDA-approved prodrugs that utilize MMAE, we employed the same self-immolative linker to bridge the pro-moiety sequence with the cytotoxic cargo (Fig. 1c).^30,31^ After building the peptide by solid-phase synthesis, including a morpholine cap to increase stability, it was coupled with the self-immolative linker para-aminobenzyl alcohol (PABC) that we had previously used in both our luminescent probe and ARV-771 prodrug.^23,25^ This provided an optimal position to couple the secondary amine of MMAE, generating our MMAE prodrug.

To mechanistically evaluate substrate engagement and release, we also generated a control prodrug in which the terminal Trp was replaced with Ala (morph-ATMA-Linker-MMAE). Previous structural studies of the immunoproteasome have revealed that the β5i subunit contains an extended, hydrophobic S1 pocket that preferentially accommodates bulky, aromatic residues and forms key π-stacking and van der Waals contacts essential for binding and catalysis. Reports comparing β5i and β5 structures further show that this pocket is both deeper and more hydrophobic in β5i, enabling selective engagement by aromatic substrates (Supplementary Fig. S2).^14,21,28^ In the context of ATMW, the substitution of Ala eliminates both steric complementarity and aromatic interactions, yielding a substantially weaker binding pose and diminished catalytic efficiency. Consistent with this rationale, ATMA-based constructs are expected to exhibit markedly reduced processing and, therefore, lower MMAE release (Supplementary Scheme S2). Although MMAE has been clinically validated as an effective prodrug cargo, we also generated an acetylated derivative that blocks the amine of MMAE rendering it ineffective to demonstrate that coupling to that position is essential in eliminating its highly toxic nature in healthy cells not expressing iCP.

With our prodrugs synthesized, we first aimed to assess the ability of the iCP to uncage and release MMAE in a biochemical setting. We have previously demonstrated our LCMS cleavage assay in the development of iCP probes and prodrugs, and utilized this method to visualize cleavage of our MMAE prodrug.^23,25,27^ Briefly, m-ATMW-PABC-MMAE is incubated with purified iCP or sCP for 1 h and then is quenched with acetonitrile. The sample is then injected onto the LCMS, and the mass of free MMAE can be extracted. From this, we were successful in identifying free MMAE, indicating the iCP can interact with our prodrug and cleave the cargo from our peptide (Fig. 1d). The prodrug that was incubated with sCP had about a fourfold reduction in MMAE cleaved, aligning with our previous selectivity reports for ATMW-containing cargo, and further validates selective activation with purified proteasomes (Fig. 1e).^29^ We then tested the ability of the iCP to cleave our control m-ATMA-PABC-MMAE prodrug and saw a reduction in the amount of MMAE that was cleaved, indicating the ATMW peptide is the preferred sequence that is required to have significant release of our compound (Fig. 1e and Supplementary Figs. S3a). The small amount of MMAE that was released by the ATMA peptide is not concerning to us because of the high amounts of iCP that are not relevant to a cellular setting, but the trend of it not cleaving as efficiently was promising to us and granted further evaluation in an endogenous environment. Before performing cellular studies, we assessed the stability of our prodrug in mouse microsomes and human serum. Using our LCMS assay, we incubated our prodrug with the pooled liver microsomes or 10% human serum at different time points before quenching and injecting it into the LCMS. From this, we could determine the amount of degraded prodrug, which remained ~80% intact after 1 h (Supplementary Fig. S3b, c).

### The immunoproteasome prodrug is active in diseased cells but inactive in healthy cells

**Fig. 2:**
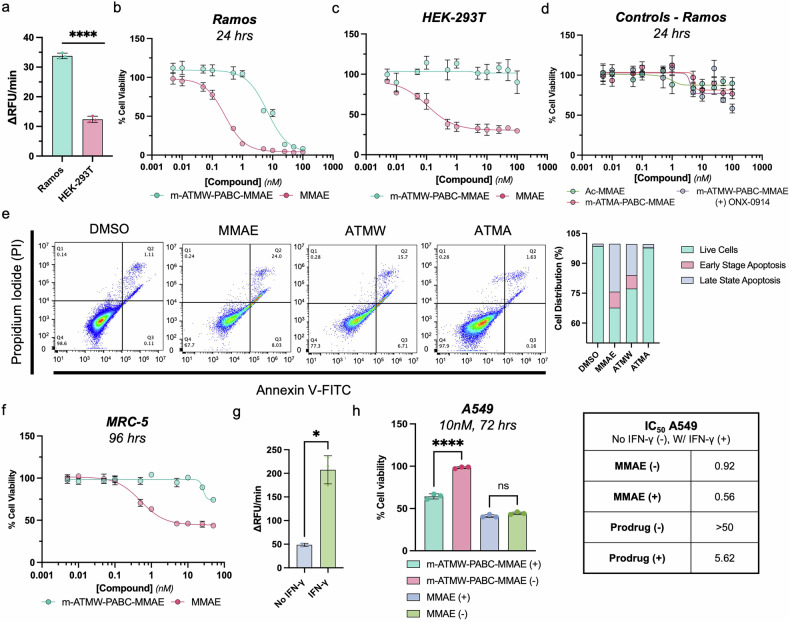
Exploring prodrug activation in cancerous vs healthy cells. **a** Activity-based assay using TBZ-1 immunoproteasome-selective probe in Ramos and HEK-293T cells. Readings taken every 90 s for 90 min with rate of cleavage calculated as ∆relative fluorescence units/time. **b** Toxicity of prodrug and free MMAE in Ramos B cells after 24 h. **c** Toxicity of prodrug and free MMAE in HEK-293T cells after 96 h. **d** Toxicity of controls m-ATMA-PABC-MMAE, Ac-MMAE, or pre-treated with iCP inhibitor ONX-0914 (10 µM for 1 h) in Ramos B cells after 24 h. **e** Apoptosis analysis by flow cytometry using Annexin V and Propidium Iodide staining in Ramos B cells after dosing with 25 nM of compound for 6 h. **f** Toxicity of prodrug or free MMAE in MRC-5 lung fibroblasts after 96 h. **g** Activity-based assay using TBZ-1 immunoproteasome selective probe in A549 cells after exposure to 5 ng/mL IFN-γ for 72 h. **h** Toxicity of prodrug or free MMAE (10 nM) in A549 cells with (+) and without (−) pre-exposure to IFN-γ. *n* = 3 per group, data are represented as mean $\pm \,$SEM

Moving away from a biochemical setting, we wanted to establish the toxicity profile of our prodrugs in a cell line that had high iCP activity and a cell line that had low iCP activity. We have already established Ramos as having high amounts of iCP and HEK-293T as having low amounts of β5i by western blot, but we wished to determine if that correlated with its activity. Using our established iCP activity-based probe, TBZ-1, we determined that Ramos had significantly higher β5i activity compared to HEK-293T cells (Fig. 2a and Supplementary Fig. S4a).^27^ If our prodrug worked as we expected, then we hoped to see that our prodrug would retain the toxicity of MMAE in the Ramos cell line, while the toxicity would be diminished in HEK-293T. Initially, using the Ramos cells that have high iCP expression, we dosed MMAE and m-ATMW-PABC-MMAE for 24 h and validated that our prodrug was able to retain single-digit nM IC_50_ values, comparable to those of the parent compound (Fig. 2b). This provided confirmation that using the iCP as a prodrug release enzyme has the capability to be used at therapeutically relevant concentrations. To ensure our peptide was not inherently toxic to the cells, leading to the significant toxicity in Ramos cells, we dosed at the same relevant concentration range in HEK-293T cells and saw no toxicity of our prodrug even at the highest concentrations for up to 96 h (Fig. 2c). MMAE remained toxic to these cells; however, its toxicity could be eliminated by being appended to our iCP selective peptide. To ensure the toxicity profiles were due to interactions with the iCP, and not different proteases specific to those cell lines, we dosed the Ramos cells with our control m-ATMA-PABC-MMAE and Ac-MMAE prodrugs that should have weaker interactions with the iCP and no longer be able to engage with the tubulin polymerization process, respectively. After treatment with these control compounds for 24 h at the relevant concentrations, the cells remained >90% viable, a stark comparison to what was observed with the actual molecules (Fig. 2d). In addition, Ramos cells were pre-dosed with the immunoproteasome-selective inhibitor ONX-0914 at 10 µM for 1 h prior to treatment with the prodrug m-ATMW-PABC-MMAE.^20^ Pre-treatment with ONX-0914 resulted in a significant reduction in cytotoxicity at relevant concentrations compared to untreated cells, consistent with immunoproteasome-dependent activation of the prodrug. At the highest concentrations of m-AMTW-PABC-MMAE, toxicity begins to re-emerge, as expected due to the combined toxicities of both the prodrug and ONX-0914. This further confirmed that the toxicity associated with our prodrug was due to the interactions it had with the iCP, leading to the release of MMAE in the β5i expressing cell line.

To further understand the mechanism of action of our prodrug in eliciting toxicity in the Ramos cell line, we performed Annexin V/Propidium Iodide (PI) staining to determine the percentage of cells undergoing apoptosis. As MMAE is a tubulin polymerization inhibitor, it elicits cell death by interfering with tubulin dimers, leading to the cell undergoing apoptosis. If our prodrug is working by releasing MMAE to elicit its mechanism of action, we expected to see a similar apoptotic profile between our prodrug and free MMAE. Ramos cells were dosed with MMAE, m-ATMW-PABC-MMAE, control m-ATMA-PABC-MMAE, or DMSO and allowed to incubate for 6 h before staining with Annexin V and PI and analyzing the cell populations by flow cytometry. From this, we were able to observe that cells dosed with DMSO or the ATMA control prodrug had no significant apoptotic signatures, while cells dosed with free MMAE or ATMW prodrug had similar shifts into early and late-stage apoptosis (Fig. 2e). This gave us confidence that the toxicity we are observing is due to the prodrug interacting with the β5i subunit, uncaging MMAE, and releasing it to perform its intended function. Next, we were interested in continuing to explore the therapeutic window of our iCP prodrug. MRC5 lung fibroblasts had previously been identified to have lower amounts of iCP compared to Ramos, and thus we wished to assess if the toxicity of our prodrug could be alleviated in this cell line as well. Upon dosing MRC5 cells with our ATMW prodrug or free MMAE for 96 h, our prodrug remained >80% viable, while cells dosed with MMAE had a reduction by over 50% in viability (Fig. 2f). We believe that the cells dosed with free MMAE not reaching 100% cell death is due to the mechanism of action of MMAE and this cell line not being as sensitive to this treatment. However, the difference in the toxicity profiles between our prodrug and MMAE still demonstrates our prodrug is capable of alleviating toxicities in a healthy cell state as compared to MMAE. Lastly, we wished to establish if our prodrug is reliant on iCP activity for release. If so, then a cell line that has inducible β5i expression should lead to an increase in the toxicity of our prodrug, while the toxicity of MMAE remains the same. To do this, we relied on the A549 cell line, and its inducible expression upon treatment with 5 ng/mL of IFN-γ for 72 h. After treatment, we initially explored the increase in β5i activity using the same TBZ-1 probe that was utilized previously.^22^ Upon treatment with 5 ng/mL for 72 h, we observed a 4-fold increase in β5i activity in the A549 cell line, establishing it as an excellent model for exploring the release of our prodrug (Fig. 2g and Supplementary Fig. S4b). Moving into toxicity characterization, we initially dosed cells with (+) or without (−) 5 ng/mL of IFN-γ for 72 h. The cells were then plated into 96-well plates and dosed with free MMAE or m-ATMW-PABC-MMAE and allowed to incubate for an additional 72 h before assessing viability. The cells that were dosed with MMAE, whether exposed to IFN-γ or not, had essentially the same toxicity profile (IC_50_ of 0.92 nM (−) vs 0.56 nM (+)). This informed us that dosing with IFN-γ does not make the cells more susceptible to MMAE toxicity, so changes that we would observe in m-ATMW-PABC-MMAE toxicity could be correlated with an increase in the release of MMAE from our prodrug. The cells that were dosed with the ATMW prodrug had an increase in toxicity when exposed to IFN-γ, leading to an almost 10-fold reduction in IC_50_ values (IC_50_ of >50 nM (−) vs 5.62 nM (+)) (Fig. 2h and Supplementary Fig. S5). These results provide a solid foundation for the use of the iCP as a prodrug release enzyme that has the capabilities to be applied at therapeutically relevant concentrations and can alleviate the harsh toxicities of MMAE in healthy cells.

### Small-cell lung cancer as a model to assess preclinical prodrug translation

Having validated our iCP prodrug in a range of cell types, we next sought to evaluate its potential across a broader spectrum of cancers. For this, we submitted our prodrug to the NCI-60 cancer panel, where it was screened across a diverse array of cancer types (Supplementary Fig. S6). Analysis of the 5-concentration dose–response data revealed that some cancers, such as leukemia, were highly sensitive to our prodrug, while others, including certain renal and CNS cancers, showed weaker responses. These findings are encouraging, as they reflect the differential expression of iCP across cancers and support our goal of achieving selective cytotoxicity correlated to iCP levels, rather than indiscriminate toxicity. To further investigate whether these differences correlate with iCP expression, we compared prodrug sensitivity with publicly available mRNA levels of the immunoproteasome subunits across the NCI-60 panel (Supplementary Fig. S7). We observed trends in which cancer types with higher iCP expression were generally more responsive to the prodrug, whereas those with low iCP expression showed minimal sensitivity. These results provide further evidence that the selective cytotoxicity of our prodrug is proportional to iCP levels, supporting our goal of targeted activity rather than indiscriminate toxicity.

**Fig. 3:**
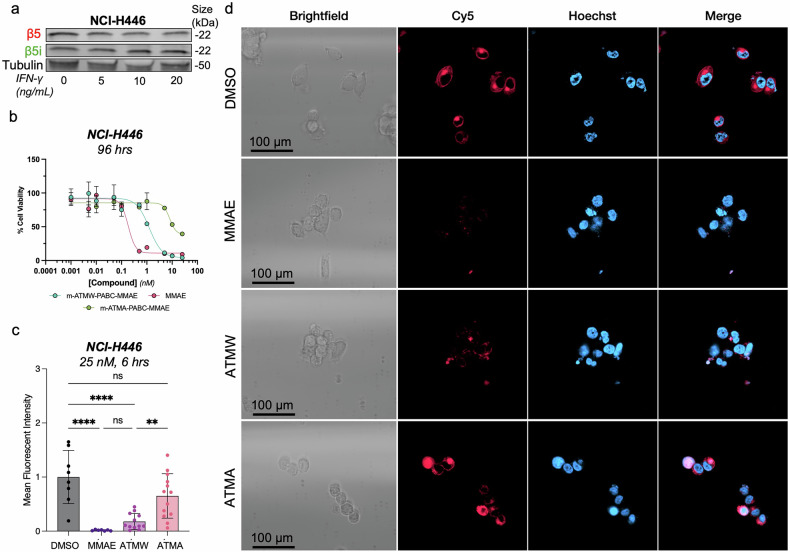
Prodrug mechanism of action in NCI-H446 small cell lung cancer model. **a** Western blot of NCI-H446 SCLC after exposure to IFN-γ. Blotting for immunoproteasome subunit β5i, standard proteasome subunit β5, and tubulin for loading control. **b** Toxicity of prodrug, free MMAE, and control prodrug in NCI-H446 after 96 h. **c** Confocal analysis of NCI-H446 cells after being dosed with 25 nM DMSO, MMAE, prodrug (ATMW), or control prodrug (ATMA) for 6 h before staining for the nucleus (blue) or tubulin (red). Magnification ×20. **d** Representative images from confocal analysis of tubulin inhibition. *n* = 3 per group, data are represented as mean $\pm \,$SEM

For the remainder of this study, we focused on small cell lung cancer (SCLC), a disease with limited treatment options and poor prognosis. SCLC accounts for ~15% of all lung cancers, with most cases diagnosed at a late stage.^32^ Even with combinations such as immune checkpoint inhibitors and chemotherapy, median survival remains around 12–15 months.^33,34^ While antibody-drug conjugates have shown some promise, identifying sufficiently overexpressed targets in SCLC has been challenging and has led to off-target toxicities.^35^ Given these limitations, SCLC presents an excellent opportunity to assess the feasibility of our iCP prodrug as a novel treatment strategy. We began by assessing the expression of β5i in the SCLC line NCI-H446. Immunoblotting for β5i revealed that NCI-H446 cells have relatively high basal levels of iCP (Fig. 3a). Treatment with iFN-γ resulted in a slight increase in β5i, supporting the suitability of this cell line for further assessment of our iCP prodrug.

We next evaluated the toxicity profile of our prodrug in this cell line. Cells were treated for 96 h with MMAE, m-ATMW-PABC-MMAE, and the control m-ATMA-PABC-MMAE, and viability was measured. Both MMAE and the iCP prodrug demonstrated potent toxicity with low nanomolar or sub-nanomolar IC_50_ values, while the control peptide was notably less toxic (Fig. 3b). Some toxicity was observed at the highest concentrations of the control peptide, likely due to the minimal change (Trp->Ala) leaving the other three residues able to interact with β5i, leading to some prodrug activation over the long incubation period. To establish the mechanism of action of our prodrug, we performed Annexin V/PI staining following treatment with DMSO, m-ATMW-PABC-MMAE, m-ATMA-PABC-MMAE, or MMAE. Both DMSO and the ATMA prodrug showed no evidence of apoptosis, while the ATMW prodrug and MMAE induced early-stage apoptosis, consistent with effective cytotoxic action (Supplementary Fig. S8). To confirm that this toxicity resulted from expected MMAE-mediated tubulin inhibition, we utilized microscopy to evaluate tubulin structure after treatment. Both MMAE and the ATMW prodrug resulted in nearly complete inhibition of tubulin, while DMSO and ATMA had no effect (Fig. 3c, d).

**Fig. 4:**
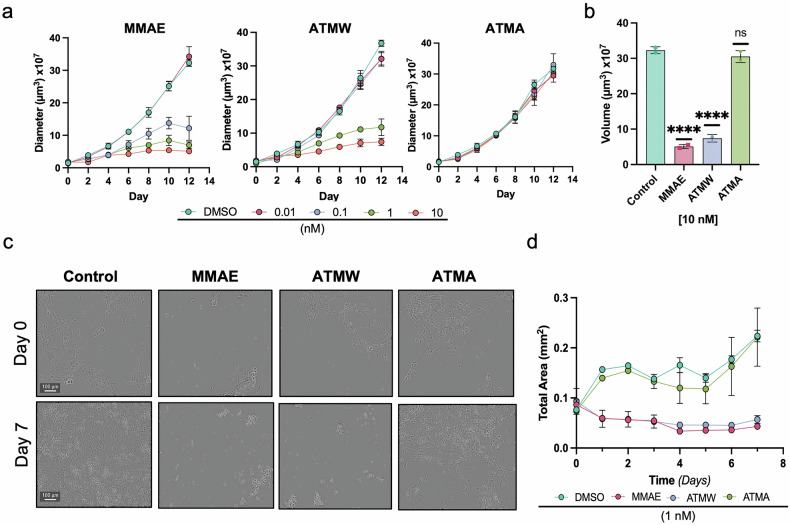
Prodrug limits the growth of SCLC cells in vitro. **a** Volume of NCI-H446 spheroids after treatment with MMAE, prodrug (ATMW), or control prodrug (ATMA) over 12 days. Images taken every day to measure volume and plotted in GraphPad Prism 10. **b** Volume of spheroids on day 12 after dosing with 10 nM free MMAE, prodrug (ATMW), or control prodrug (ATMA). **c** Representative images of cell growth analysis of NCI-H446 on day 0 and day 7 after dosing with 1 nM free MMAE, prodrug (ATMW), or control prodrug (ATMA). **d** Total area calculated for wells of cell growth analysis after dosing with 1 nM free MMAE, prodrug (ATMW), or control prodrug (ATMA). *n* = 2 per group, data are represented as mean $\pm \,$SEM

With confidence in our iCP prodrug mechanism, we sought further validation in more complex models. Using low-adhesion plates, NCI-H446 cells were grown as 3D spheroids to better mimic tumor architecture.^36,37^ After spheroid formation, cells were exposed to DMSO, MMAE, m-ATMW-PABC-MMAE, or m-ATMA-PABC-MMAE and imaged daily for 12 days (Fig. 4a and Supplementary Fig. S9). Only spheroids treated with MMAE or ATMW prodrug exhibited a significant reduction in spheroid size, with effects notable even at 1 nM, while DMSO and ATMA control had little impact (Fig. 4b). We also assessed NCI-H446 growth in monolayer cultures. After cells adhered, they were treated with 1 nM of each compound, and their growth was monitored over 7 days (Fig. 4c and Supplementary Fig. S10). MMAE and ATMW completely suppressed growth, while DMSO and ATMA controls had little effect (Fig. 4d). This demonstrates that prodrug activation is dependent on iCP presence.

Lastly, to confirm iCP-mediated activation, we performed siRNA knockdown of β5i in NCI-H446 cells (Supplementary Fig. S11a). Partial knockdown (~ 40%) was achieved using 10 nM siRNA without significant baseline toxicity. After knockdown, cells treated with the ATMW prodrug showed a 10-fold reduction in toxicity compared to non-silenced controls, while MMAE-induced toxicity was unchanged by β5i knockdown (Supplementary Fig. S11b). These findings further confirm that prodrug activation and cytotoxicity are mediated by iCP.

### An immunoproteasome prodrug can reduce the toxicity of MMAE in vivo

Up to this point, all data supporting iCP-mediated prodrug activity have been generated through biochemical or cell-based assays. To further explore the translational potential of this new approach, we aimed to assess its effectiveness in vivo. Since this would be the first time an iCP prodrug has been tested in mice, our goal was to determine whether the prodrug could mitigate the well-known toxicities associated with free MMAE administration. Demonstrating improved tolerability alone would provide a strong rationale for further pursuing this strategy as a novel cancer treatment.

**Fig. 5:**
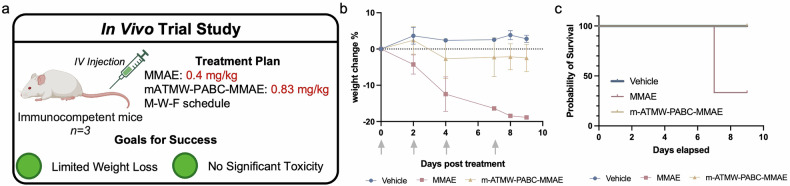
In vivo toxicity pilot study. **a** Overall plan for the pilot study. Immunocompetent mice bearing SCLC tumors (*n* = 3) are treated with MMAE (0.4 mg/kg), m-ATMW-PABC-MMAE (0.83 mg/kg), or Vehicle on a Monday, Wednesday, Friday schedule. In total, 0.4 mg/kg of MMAE was chosen as this dose is known to elicit significant toxicities shortly after IV administration. In total, 0.83 mg/kg of our prodrug is the same effective concentration of MMAE and would provide information about the safety of using our prodrug to limit unwanted toxicities. **b** Weight loss analysis after mice dosed with prodrug (0.8 mg/kg), free MMAE (0.4 mg/kg), or vehicle (*n* = 3). Mice dosed on days indicated by gray arrows. **c** Probability of survival after dosing with prodrug (0.8 mg/kg), free MMAE (0.4 mg/kg), or vehicle (*n* = 3)

Given that MMAE is highly toxic in mice, we initially conducted a pilot study to assess whether our prodrug could reduce MMAE-related toxicities in a small group of immunocompetent mice.^38,39^ It is well established that uncaged MMAE will cause toxicity and weight loss in mice after being exposed to one dose at 0.4 mg/kg.^40^ For this pilot study, mice were randomly assigned to one of three treatment groups (*n* = 3 per group): Vehicle (0 mg/kg), MMAE (0.4 mg/kg), and m-ATMW-PABC-MMAE (0.83 mg/kg, equivalent to the MMAE dose). Mouse-derived SCLC cells were injected orthotopically into the lungs and could be used to assess any reduction in tumor volume from our prodrug. Treatments were administered on a Monday, Wednesday, Friday schedule for 9 days (Fig. 5a). Mouse weight and behavior were monitored every other day to track signs of safety and toxicity. Within 4 days, the MMAE group showed rapid weight loss and severe toxicity (with only one mouse remaining), while both the vehicle and prodrug groups maintained stable weights (Fig. 5b). The study continued until day 9, when the final mouse in the MMAE group succumbed to toxicity. Notably, all mice treated with our prodrug survived dosing at the equivalent effective MMAE concentration with no significant weight loss (Fig. 5c). In addition, bioluminescent images of the tumors showed a significant reduction after treatment with our prodrug as well as mild evidence of toxicity as determined by serum biochemistry analysis from the surviving mice (Supplementary Fig. S12). The elevated creatine kinase and BUN levels observed in m-ATMW-PABC-MMAE-treated mice may reflect tumor lysis-associated systemic effects, consistent with an on-target anti-tumor therapeutic response. These findings validate that targeting the immunoproteasome for prodrug activation can minimize MMAE toxicity in vivo and support the translational potential of this approach for cancer therapy.

### Small cell lung cancer tumor burden reduced after treatment with immunoproteasome prodrug

**Fig. 6:**
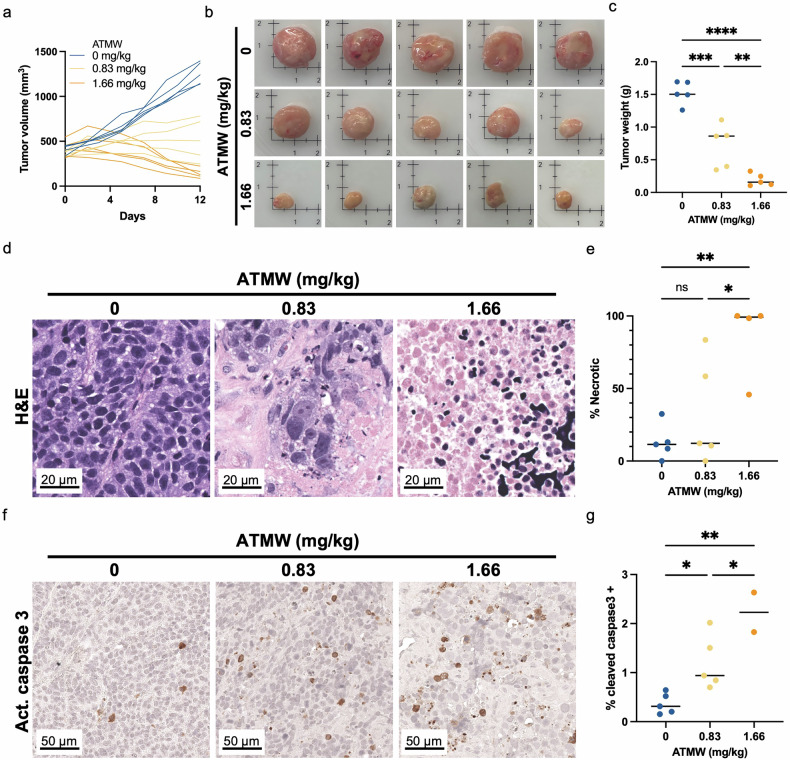
NCI-H446 SCLC tumor model. **a** Distribution of tumor volumes (mm^3^) as determined by caliper measurements taken every 2 days. Five mice per dosing group were subjected to either vehicle (0 mg/kg), low dose (0.83 mg/kg), or high dose (1.66 mg/kg) of m-ATMW-PABC-MMAE. **P = 0.0012; ****P < 0.0001 by Dunnett’s multiple comparisons test. **b** Excised tumors from individual mice at the end of the study for each dosing group. **c** Tumor weights (g) at the end of the study after excising from the mice. Each dot represents an independent mouse, and the solid line represents the mean. **P = 0.01; ***P < 0.001; ****P < 0.0001 by ordinary one-way ANOVA. **d** Representative H&E staining of tumors to signify necrosis. **e** %Necrosis as determined from H&E staining across the dosing groups. Each dot represents an independent mouse, and the solid line represents the mean. Ns not significant; *P < 0.05; **P < 0.01; ***P < 0.001; ****P < 0.0001 by ordinary one-way ANOVA. **f** Representative activated caspase 3 (brown) staining of tumors to identify the extent of apoptosis. **g** % cleaved caspase 3 from the staining of tumors across the dosing groups. Each dot represents an independent mouse, and the solid line represents the mean. *P < 0.05; **P < 0.01 by ordinary one-way ANOVA

While our initial pilot study provided the first in vivo evidence that our iCP prodrug can eliminate the off-target toxicities of MMAE, further validation in a larger study was warranted. Having established the promise of this approach, we expanded our investigation and tested whether higher doses could decrease tumor burden without increasing toxicity. In this expanded study, NSG mice were subcutaneously implanted with NCI-H446 SCLC cells to allow for accurate, consistent tumor volume measurements.^41^ Mice were divided into three treatment groups (*n* = 5 per group): Vehicle (0 mg/kg), a low dose of m-ATMW-PABC-MMAE (0.83 mg/kg), and a high dose (1.66 mg/kg). The low dose is the same amount we used in the initial trial, while the high dose would double the amount of prodrug the mice would see (2x equivalent dose of MMAE). Treatments were given on a Monday, Wednesday, and Friday schedule, and tumor volume and body weight were measured. Results showed that all mice receiving either low or high prodrug doses completed the regimen without significant weight loss or other signs of toxicity, even at the highest dose (Supplementary Fig. S13a). Both dosing groups experienced a significant reduction in tumor volume compared to the vehicle, with the high dose demonstrating near-complete tumor regression (Fig. 6a–c). Histologic evaluation of spleens from NSG mice treated with increasing doses of ATMW demonstrated preserved red pulp architecture without evidence of treatment-related toxicity. No necrosis, hemorrhage, or structural disruption of the spleen was observed at any dose level (Supplementary Fig. S13b). At the end of the study, tumor excision confirmed the observed reductions across the groups. Importantly, to verify that tumor reduction was mediated by release of the active MMAE payload, LCMS analysis of excised tumor tissue confirmed detectable levels of MMAE only in prodrug-treated tumors, demonstrating in situ activation of the iCP-targeted prodrug (Supplementary Fig. S14).

To further confirm the expected mechanism of tumor cell death, we performed H&E staining for necrosis and immunohistochemistry for activated caspase 3 (Fig. 6d–g). High-dose prodrug-treated tumors displayed significantly higher necrosis compared to both low-dose and vehicle, and both dosing groups showed enhanced caspase 3 activation, indicating apoptosis. To further understand the selectivity of our prodrug in vivo, we also performed serum biochemistry analysis and observed no noticeable toxicities, even within the mice treated at the highest concentration of prodrug (Supplementary Fig. S15). While further studies will be required to establish the maximal tolerated dose and fully define the therapeutic window, these results provide robust preclinical validation for the iCP prodrug platform and lay a solid foundation for continued exploration of iCP-targeting prodrugs as a promising cancer therapy.

### Immunoproteasome prodrug preserves B- and CD4 T-cell populations in human-derived organoids

Having established that our prodrug is both effective and has minimal toxicities to both immunocompetent and NSG mice, we were interested in understanding how our prodrug was affecting human immune cells specifically. To evaluate the impact of immunoproteasome-targeted prodrug activation in a human primary immune context, we assessed effects in tonsil-derived immune organoids.^42^ Organoids from eleven independent donors were treated with MMAE or the immunoproteasome prodrug at 10 µM, a dose selected to optimize viability while preserving phenotypic differences between compounds, and were cultured in the presence or absence of live attenuated influenza vaccine (LAIV) to induce immune activation. Cultures were harvested four days post-treatment and analyzed by high-dimensional spectral flow cytometry. Across conditions, total cell viability was largely preserved, with only minor reductions observed following MMAE treatment. Overall, B-cell and CD4 T-cell frequencies were not majorly altered by either compound. Within the B cell compartment, MMAE treatment resulted in increased frequency of pre-germinal (PreGC) B cells while concomitantly suppressing plasmablast populations. In contrast, plasmablast frequencies were preserved in prodrug-treated organoids under both unstimulated and LAIV-stimulated conditions, despite modest changes in germinal center B-cell subsets. Analysis of germinal center dynamics revealed that dark zone B-cell frequencies were increased following MMAE treatment, while remaining comparable to control conditions in prodrug-treated organoids, suggesting differential effects on proliferative or selection processes within the germinal center. Within the CD4 T-cell compartment, regulatory T cells were markedly suppressed following MMAE treatment, whereas these cells were maintained in prodrug-treated organoids. Collectively, these data demonstrate that free MMAE broadly disrupts immune cell composition and differentiation in human immune organoids, particularly under inflammatory conditions, whereas the immunoproteasome-targeted prodrug preserved key B and CD4 T-cell populations, including GC B cells, plasmablasts, and regulatory T cells (Supplementary Fig. S16). These findings indicate that immunoproteasome-dependent prodrug activation mitigates the immune-toxic effects associated with MMAE while maintaining immune responsiveness in a human primary tissue setting.

## Discussion

Targeting the proteasome with small-molecule inhibitors has been a well-validated therapeutic strategy for the past 20 years.^15^ More recently, the development of targeted protein degradation has reignited the proteasome as a target for drug discovery.^43^ Although targeting the proteolytic activity of the catalytic core particle has been advantageous in treating cancers such as multiple myeloma, off-target effects limit the overall efficacy of this therapeutic strategy.^44,45^ To overcome these challenges, targeting the immunoproteasome (iCP) with small-molecule peptidomimetics has also accelerated interest as the iCP is typically expressed in cells that are exposed to inflammatory cytokines, such as IFN-γ and TNF-α.^16,46^ Again, this direct targeting of the iCP in diseased cells has shown to be of clinical relevance in treating inflammatory diseases such as Lupus as well as has been explored for cancer.^47^ Altogether, targeting the catalytic nature of this complex fails to fully encompass the sophisticated nature of the proteasome and how it could be targeted through alternative strategies. We proposed that this large, multi-catalytic complex could be harnessed, rather than inhibited. This would take advantage of the diseased cell expression and upregulated activity of the iCP, without affecting its native immune functions. Using the iCP as a prodrug uncaging enzyme could create therapeutic strategies for a variety of inflammatory diseases including cancers where the iCP is upregulated, without affecting healthy cells.

To accomplish this, we took advantage of a recognition sequence that was discovered to be selective for the iCP over the sCP in the development of an activity-based probe.^22^ We were inspired that the activity of the iCP could be harnessed to release a large fluorophore, such as rhodamine, in cancerous cells that had high iCP activity, while no signal was achieved in healthy cells due to their lack of iCP expression. We leveraged this selectivity and replaced the fluorophore with MMAE, a highly potent chemotherapeutic widely used in FDA-approved antibody-drug conjugates (ADCs) such as Brentuximab vedotin.^26^ Unlike conventional ADCs, which rely on antibody-antigen binding, internalization, and lysosomal processing to deliver MMAE, our iCP-prodrug strategy uses intracellular protease activity as the activation trigger, thereby bypassing antigen heterogeneity and trafficking constraints that limit ADC efficacy. With this new prodrug system in place, we explored whether we could reproduce the selective activation seen in fluorogenic studies, now using toxicity as a functional readout of MMAE release. We demonstrated that our iCP prodrug was only active in cell lines with high iCP activity at low nM to pM concentrations, while no toxicity occurred in healthy models even at the highest doses for up to 96 h. We then explored small-cell lung cancer (SCLC) as a model to assess both mechanism and translational potential. Our findings validated that the prodrug acted through canonical MMAE-mediated mechanisms, highlighting that protease-based activation can achieve the potency associated with ADCs without the dependence on antigen expression or antibody engineering.

We next sought to push the limits of this therapeutic modality by performing the first in vivo assessment of iCP prodrug activation. Until now, the translational potential of harnessing iCP activity to release therapeutically active compounds was unknown. We found that our prodrug could eliminate the severe systemic toxicity associated with MMAE, as mice dosed with our prodrug survived the entire pilot study, in contrast to the rapid toxicity observed with free MMAE. Importantly, this ability to uncouple potency from systemic toxicity addresses a central limitation of ADC platforms such as Brentuximab vedotin, where premature payload release, heterogeneous drug-antibody ratios, and restricted tumor penetration can produce narrow therapeutic windows and dose-limiting toxicities. In a human SCLC xenograft model, mice dosed with our iCP prodrug at the low concentrations 0.83 mg/kg and 1.66 mg/kg showed significant tumor reduction with robust activation of apoptotic and necrotic markers. In addition, mice dosed with both concentrations of prodrug had no noticeable toxicities from serum analysis or weight loss, further confirming that the iCP-mediated prodrug activation can selectively target cancer cells in vivo while sparing healthy tissues.

Given that the iCP is expressed in immune cells, the absence of observable toxicity was an important consideration. Our cellular studies indicate that dendritic cells, although express considerable iCP, have lower iCP activity relative to our cancerous cells, and the prodrug did not exhibit cytotoxicity toward these cells in vitro (Supplementary Fig. S17). In addition, we explored prodrug treatment in human-derived tonsil-organoids to assess any immune cell toxicities in actual patient samples. These results further confirmed that our iCP prodrug was not affecting immune cells. This may reflect differences in the expression or activation of iCP regulators, or a lower reliance on iCP activity for proteolysis in immune cells compared to highly proliferative cancer cells. While additional studies will fully characterize immune cell sensitivity and cytokine responses, these initial findings are consistent with the lack of systemic toxicity observed in vivo and suggest that immune cells are less susceptible to iCP-targeted prodrug activation under the conditions tested.

This provides the first demonstration that iCP-targeted prodrugs can be effective in a preclinical model of cancer. While this report focuses on SCLC, our NCI-60 data and corresponding mRNA levels of iCP suggest broad utility in diverse cancers, especially ones where ADCs have typically been ineffective, such as triple-negative breast cancer. We anticipate that this protease-responsive strategy will stimulate new drug discovery efforts targeting proteasome isoforms across disease contexts. Moreover, because our approach does not rely on antibody conjugation for delivery or activation, it offers a tractable alternative in indications where antigen-directed ADCs are ineffective, unavailable, or hindered by poor tumor penetration, high heterogeneity, or manufacturing complexity.

## Materials and methods

### General methods and materials

All chemicals, reagents, and solvents were purchased from commercial vendors and were used as such. Reactions were monitored by LCMS on an Agilent 1260 Infinity II system with a Single Quadrupole LC/MS system. HRMS was performed by UCI’s Mass Spectrometry Core facility. Mass of the synthesized molecule was recorded using an Agilent 1260 series LC coupled to an ESI-MS detector. Purification was performed on an Agilent 1260 Infinity II Analytical HPLC reverse phase with scalar (c18) column. 20S Human Immunoproteasome (SBB-PP0004) and 20S Human Proteasome (SBB-PP0005) was purchased from South Bay Bio.

### General solid phase synthesis of m-AT(tBu)MW(Boc) (1)

Peptide m-ATMW was synthesized by solid-phase peptide synthesis (SPPS). 2-Chlorotrityl chloride resin (1.0–1.2 meq/g, 200–400 mesh, Chem-Impex Int’l Inc.) was swelled in dry dichloromethane (DCM) for 60 min at room temperature (RT). Fmoc-Trp(boc)-OH (2 equiv relative to resin) and diisopropylethylamine (DIPEA) (4 eq. relative to resin) were dissolved in dry DCM (10 mL/g of resin) and added to the resin. Resin was agitated with this mixture for 60 min at RT. Then, a DCM/methanol/DIPEA (17:1:2) mixture was added to the resin and agitated for 60 min at RT to cap any unreacted trityl sites. Resin was washed with DCM (3×) and then dimethylformamide (DMF) (3×). Fmoc deprotection was done with 20% piperidine in DMF for 15 min at RT. Fmoc-Met-OH (5 equiv), HBTU (4.5 equiv), and DIPEA (10 equiv) in DMF were added to the resin. Resin was agitated with this reaction mixture for 60 min at RT. Fmoc deprotection was done with 20% piperidine in DMF for 15 min at RT. Fmoc-Thr(tBu)-OH (5 equiv), HBTU (4.5 equiv), and DIPEA (10 equiv) in DMF were added to the resin. Resin was agitated with this reaction mixture for 60 min at RT. Lastly, Fmoc-Ala-OH (5 equiv), HBTU (4.5 equiv), and DIPEA (10 equiv) in DMF were added to the resin. Fmoc deprotection was done with 20% piperidine in DMF for 15 min at RT. The resin was washed with DMF (3×) and then with dry DMF (3×). Lastly, the *N-*terminus was capped with a morpholine group. Bromoacetic acid (2 M) and DIC (1 M) were prepared in dry DMF and allowed to react for 5 min at 37 °C. The activated mixture was added to the resin and reacted for 20 min at 37 °C. The solution was drained and subsequently added morpholine (0.5 M) in dry DMF and allowed to react for 1 h at 37 °C. Completion of the morpholine cap was checked by the Kaiser test. m-AT(tBu)MW(Boc) resin was washed with DCM, suspended in strong cleavage cocktail (2.5% TFA, 95% DCM, and 2.5% triisopropylsilane), and agitated for 60 min at RT. The cleavage mixture was then collected and evaporated with argon. Crude m-AT(tBu)MW(Boc) was precipitated with cold ether and centrifuged. Ether supernatant was decanted, and the crude peptide was washed with ether (2×). The powder was dried under vacuum overnight.

### Synthesis of m-AT(tBu)MW(Boc)-PABA (2)

m-AT(tbu)MW(boc) (1 equiv.), 4-aminobenzyl alcohol (1.2 equiv.), and n-Methyl Imidazole (3.1 eq) were dissolved in DCM before addition of Chloro-*N*,*N*,*N*′,*N*′-tetramethylformamidinium hexafluorophosphate (TCFH) (1.1 equiv). The reaction was stirred at r.t. overnight. Solvent removed under vacuum, water added, and extracted with ethyl acetate 3×. The organic layer was washed with sat. sodium bicarbonate 3×, brine 3×, then dried with MgSO_4_ and solvent removed under vacuum. The crude product was subsequently used in the next reaction without purification.

### Synthesis of m-AT(tBu)MW(Boc)-PABC (3)

m-AT(tBu)MW(Boc)-PABA (**2**) (1 equiv.) was dissolved in THF before the addition of Bis(4-nitrophenyl)carbonate (3 equiv.) and DIPEA (6 equiv.). The solution was allowed to stir overnight at r.t. Upon completion of the reaction as determined by LCMS, THF removed in vacuum, and the product was redissolved in ethyl acetate. Organic layer washed with sat. sodium bicarbonate 3×, brine 3×, then dried with MgSO_4_ and solvent removed under vacuum. Product redissolved in 50/50 Water/ACN (0.1% TFA) for HPLC purification (yield: 25% overall).

### Synthesis of m-AT(tBu)MW(Boc)-PABC-MMAE (4)

m-AT(tBu)MW(Boc)-PABC (**3**) was dissolved in DMF before the addition of MMAE (2 equiv.), DIPEA (4 equiv), and Pyridine (2 equiv). Solution was allowed to stir for 48 h at r.t. Reaction monitored by LCMS, and once all (**3**) was gone sat. citric acid was added, and ethyl acetate was used to extract (3×). Organic layer washed with sat. sodium bicarbonate 3×, brine 3×, then dried of MgSO_4_ and solvent removed under vacuum.

### Synthesis of m-ATMW-PABC-MMAE (5)

m-AT(tBu)MW(Boc)-PABC-MMAE (**4**) redissolved in 95% TFA, 2.5% DCM, 2.5% TIPS, and allowed to stir for 1 h at r.t. After stirring, the TFA solution was blown off with argon and the product redissolved in 50/50 Water/ACN (0.1% TFA) for HPLC purification. (Yield: 7%) HRMS: Theoretical [M+Na]^+^ 1505.8094 + /− 3.0 mDa. Observed: 1505.8085, −0.9 mDa.

### Synthesis of control peptide AT(tbu)MA (6)

Peptide m-ATMW was synthesized by solid-phase peptide synthesis (SPPS). 2-Chlorotrityl chloride resin (1.0–1.2 meq/g, 200–400 mesh, Chem-Impex Int’l Inc.) was swelled in dry dichloromethane (DCM) for 60 min at room temperature (RT). Fmoc-Ala-OH (2 equiv relative to resin) and diisopropylethylamine (DIPEA) (4 eq. relative to resin) were dissolved in dry DCM (10 mL/g of resin) and added to the resin. Resin was agitated with this mixture for 60 min at RT. Then, a DCM/methanol/DIPEA (17:1:2) mixture was added to the resin and agitated for 60 min at RT to cap any unreacted trityl sites. Resin was washed with DCM (3×) and then dimethylformamide (DMF) (3×). Fmoc deprotection was done with 20% piperidine in DMF for 15 min at RT. Fmoc-Met-OH (5 equiv), HBTU (4.5 equiv), and DIPEA (10 equiv) in DMF were added to the resin. Resin was agitated with this reaction mixture for 60 min at RT. Fmoc deprotection was done with 20% piperidine in DMF for 15 min at RT. Fmoc-Thr(tBu)-OH (5 equiv), HBTU (4.5 equiv), and DIPEA (10 equiv) in DMF were added to the resin. Resin was agitated with this reaction mixture for 60 min at RT. Lastly, Fmoc-Ala-OH (5 equiv), HBTU (4.5 equiv), and DIPEA (10 equiv) in DMF were added to the resin. Fmoc deprotection was done with 20% piperidine in DMF for 15 min at RT. The resin was washed with DMF (3×) and then with dry DMF (3×). Lastly, the *N-*terminus was capped with a morpholine group. Bromoacetic acid (2 M) and DIC (1 M) were prepared in dry DMF and allowed to react for 5 min at 37 °C. The activated mixture was added to the resin and reacted for 20 min at 37 °C. The solution was drained and subsequently added morpholine (0.5 M) in dry DMF and allowed to react for 1 h at 37 °C. Completion of the morpholine cap was checked by the Kaiser test. m-AT(tBu)MW(Boc) resin was washed with DCM, suspended in strong cleavage cocktail (2.5% TFA, 95% DCM, and 2.5% triisopropylsilane), and agitated for 60 min at RT. The cleavage mixture was then collected and evaporated with argon. Crude m-AT(tBu)MW(Boc) was precipitated with cold ether and centrifuged. Ether supernatant was decanted, and the crude peptide was washed with ether (2×). The powder was dried under vacuum overnight.

### Synthesis of m-AT(tBu)MA-PABA (7)

m-AT(tbu)MA (1 equiv.), 4-aminobenzyl alcohol (1.2 equiv.), and n-Methyl Imidazole (3.1 eq) were dissolved in DCM before addition of Chloro-*N*,*N*,*N*′,*N*′-tetramethylformamidinium hexafluorophosphate (TCFH) (1.1 equiv). The reaction was stirred at r.t. overnight. Solvent removed under vacuum, water added, and extracted with ethyl acetate 3×. The organic layer was washed with sat. sodium bicarbonate 3×, brine 3×, then dried with MgSO_4_ and solvent removed under vacuum. The crude product was subsequently used in the next reaction without purification.

### Synthesis of m-AT(tBu)MA-PABC (8)

m-AT(tBu)MA-PABA (**7**) (1 equiv.) was dissolved in THF before the addition of Bis(4-nitrophenyl)carbonate (3 equiv.) and DIPEA (6 equiv.). The solution was allowed to stir overnight at r.t. Upon completion of the reaction as determined by LCMS, THF was removed in vacuum, and the product was redissolved in ethyl acetate. Organic layer washed with sat. sodium bicarbonate 3×, brine 3×, then dried with MgSO_4_ and solvent removed under vacuum. Product redissolved in 50/50 Water/ACN (0.1% TFA) for HPLC purification.

### Synthesis of m-AT(tBu)MA)-PABC-MMAE (9)

m-AT(tBu)MA-PABC (**8**) was dissolved in DMF before the addition of MMAE (2 equiv.), DIPEA (4 equiv), and Pyridine (2 equiv). Solution was allowed to stir for 48 h at r.t. Reaction monitored by LCMS, and once all (**3**) was gone sat. citric acid was added, and ethyl acetate was used to extract (3×). Organic layer washed with sat. sodium bicarbonate 3×, brine 3×, then dried over MgSO_4_ and solvent removed under vacou.

### Synthesis of m-ATMA-PABC-MMAE (10)

m-AT(tBu)MA-PABC-MMAE (**9**) redissolved in 95% TFA, 2.5% DCM, 2.5% TIPS, and allowed to stir for 1 h at r.t. After stirring, the TFA solution was blown off with argon and the product redissolved in 50/50 Water/ACN (0.1% TFA) for HPLC purification. HRMS: Theoretical [M + 2H/2]^2+^ 684.8960 + /− 3.0 mDa. Observed: 684.8980 + 2 mDa.

### Synthesis of Ac-MMAE

MMAE (1 equiv.) was dissolved in THF before the addition of acetic anhydride (2 equiv.) and DIPEA (3 equiv.). The reaction was left to stir for 2 h at r.t. TLC was used to monitor reaction conversion. After the product had formed, the THF was removed under vacuum before redissolving in 25 mL of DCM and washing with citric acid. The organic layer was then washed with saturated sodium bicarbonate 3×, brine 3×, and dried over MgSO_4_, and solvent removed under vacuum. Redissolved in 50/50 Water/ACN (0.1% TFA) for HPLC purification. HRMS: Theoretical [M + H] 760.4, Observed 760.4.

### Biochemical LC/MS cleavage analysis

Each compound was initially dissolved in DMSO and further diluted in 50 mM Tris HCl, pH 7.5, at a proper stock concentration to achieve a final DMSO concentration of 2% in the 50 μL biochemical assay. In a black flat-bottom 96-well plate, the compound was incubated with either buffer human 20S proteasome, 5 nM of human 20S immunoproteasome. After the designated time point, the 20S cleavage reaction was quenched by adding 50 μL acetonitrile. Samples were dried with a speed vacuum and then reconstituted in 105 μL of 50/50 water/acetonitrile with 0.1% formic acid.

In total, 100 μL of each sample was run on an Agilent 1260 Infinity II with a Zorbax StableBond-C18 1.8 micron, 2.1 × 50mm, column attached to an Agilent 6129 quadrupole mass spectrometer. The column was held at 30 °C. Flow rate was set at 0.450 mL/min. Mobile solution A was 0.1% formic acid in water, and mobile phase B was 0.1% formic acid in acetonitrile. The gradient used was held at 5% B for 2 min, increased linearly to 100% B for 8 min, and then held at 95% B for 2 min. The mass data was scanned in positive ion mode and collected at a range of 300–900 *m/z* in starting 2 min after injection. MSD’s spray chamber was set to API-ES (atmospheric pressure ionization–electrospray ionization), with drying gas flow at 12.0 L/min, nebulizer pressure at 35 psig, drying gas temperature at 300 °C, and positive capillary voltage at 3000 V. For each 20S-treated sample, the mass of free MMAE was extracted (718 ± 0.5 Da), and the area under the curve was measured. To calculate the percent release of MMAE, a standard curve of free MMAE from 0.05 to 10 μM prepared in 50 mM Tris-HCl buffer. The solutions were diluted by adding 50 μL acetonitrile. Samples were dried with a speed vacuum and then reconstituted in 105 μL of 50/50 water/acetonitrile with 0.1% formic acid.

### Time-dependent serum stability of prodrug

The compound was initially dissolved in DMSO and then further diluted in a 50 mM Tris HCl, pH 7.5 buffer, at a proper stock concentration to achieve a final DMSO concentration of 2% in 50 µL total for the biochemical assay. In a polypropylene 96-well plate, the compound was incubated with buffer (50 mM Tris HCl at pH 7.5), or 10% human serum in buffer at 37 °C for the desired time points. Once the time point was reached, the proteins from the serum were precipitated by adding chilled acetonitrile (such that the total volume was ~85% acetonitrile). Samples were then vortexed and stored at −20 °C for at least 2 h. Samples centrifuged at 21,000 × *g* for 30 min at 4 °C. In total, 300 µL of the supernatant was carefully removed and transferred to a fresh Eppendorf tube. Samples were dried with a speed vacuum and then reconstituted in 105 µL of a mixture of water and acetonitrile with 0.1% formic acid.

### Time-dependent microsomal stability of prodrug

Initially, a 100 mM potassium phosphate buffer was prepared, pH 7.5 in 1 L of DI water. The compounds were initially dissolved in DMSO to make a 20 mM stock. From the stock, 0.5 µL was further diluted to 10 µL in DMSO. This was added to 1000 µL of the previously prepared potassium phosphate buffer. 125 µL of microsomes were diluted in 3875 µL of warmed buffer.

After preparing all of the reagents, 25 µL of compound was added to a 96-well black plate and placed in an incubator to warm to 37 °C. 200 µL of the microsome solution was added to all wells. NADPH was diluted in 1 mL of water to make a 10 mM solution (make this fresh right before adding to samples). In total, 25 µL of the NADPH solution was added to the wells, and buffer was added to control wells with just microsomes. Overall, 50 µL of the sample is taken and quenched with acetonitrile to serve as *T* = 0. Every 20 min another 50 µL sample is taken and quenched with acetonitrile over the course of an hour. Samples are spun down at 3000 × *g* for 15 min at 4 °C. In total, 50 µL of the sample is taken and diluted with a further 50 µL of HPLC-grade water. In all, 90 µL injected onto LCMS and analyzed by extracting for mass corresponding to the intact prodrug to determine % remaining over time.

### General cell culture

Ramos (ATCC® CRL-1596™), were grown in RPMI-1640 (ATCC® 30-2001™) supplemented with 10% FBS. A549 (ATCC® CCL-185) were grown in F12K (ATCC® 30-2004™) supplemented with 10% FBS, MRC-5 (ATCC® CCL-171) were grown in EMEM (ATCC® 30-2003) supplemented with 10% FBS and 1× penicillin/streptomycin (PEST), HEK-293T (ATCC® CRL-3216) were grown in DMEM (ATCC® 30-2002) supplemented with 10% FBS, NCI-H446 (ATCC HTB-171). All cells were incubated at 37 °C with 5% CO_2_. For suspension cells, the cell media was pelleted @ 800×*g* for 5 min. For adherent cells, the media was aspirated, and the flask rinsed with sterile PBS before treating cells with 0.25% (w/v) Trypsin-0.53 mM EDTA solution. The flasks were returned to the incubator for 5 min. Trypsin was quenched with supplemented media, suspension was collected, and cells were pelleted @ 1000 rpm for 5 min. After aspirating media, the cell pellet was resuspended in fresh media with 10% FBS. Cell concentration was determined by Trypan Blue, a hemocytometer, and plated accordingly.

### IFN- γ-treated cells

For LMP7/PSMB5 western blots: Cells were seeded in a six-well (300,000 cells/well) and exposed to 0, 5, 10, and 20 ng/mL of IFN-γ for 72 h with daily dosing. After the 72 h time point, cells were collected and lysed with RIPA with 1× HALT. Cells were allowed to incubate with lysis buffer for 10 min before being subjected to centrifugation at 14,000×*g* for 15 min. Supernatant collected and protein normalized by BCA assay. In total, 30 ng of protein loaded into 10-well gels w/ 1× Laemmli loading buffer and BME. Gel ran at 175 V for ~40 min or until the bottom of the gel ran off. Gels were transferred using the Trans-Blot Turbo transfer method. Membranes were blocked with 50% blocking buffer and PBS for 1 h before incubating with Anti-LMP7, Anti-PSMB5, and Anti-Alpha Tubulin overnight at 4 °C. Primaries were removed and incubated with secondary (anti-goat, anti-rabbit, and anti-mouse) antibodies for 1 h at RT. Membrane imaged on Sapphire 600 in the 800 and 680 channels. Bands normalized to tubulin, the amount of LMP7 and PSMB5 was determined.

For toxicity studies: Cells were seeded in a T-25 (1.4 × 10^6^ cells). Daily dosing of 20 ng/mL of IFN-γ or DMSO to the control group for 72 h. On the 48 h mark, cells were collected and counted before seeding in a 96-well white plate at 20,000 cells/well (with 20 ng/mL or DMSO for the 72 h time point). After the time point, cells were dosed with the compound as described below; no additional IFN-γ was added at this time.

### Cell toxicity with adherent cells

Cells were plated in 96-well opaque plates at a density of 20,000 cells/well (100 µL per well of a 200,000 cells/mL stock). Overall, 200 µL of PBS was added to the surrounding wells. Cells were left to adhere overnight in the incubator. After adherence, media was removed, and cells were dosed with 100 µL 1× target concentration of compound stocks prepared at 0.5% DMSO solution in cell media (1% FBS) in triplicate. Cell was placed into the incubator for 24 or 96 h time incubation. After time point, 90 µL Cell Titer-Glo® luminescent reagent was added to each well. The plate was gently shaken for 2 min. The plate was then protected from light for 10 min prior to being measured. Luminescence was recorded using a BioTek Synergy™ Neo2 Multimode Microplate Reader with a gain of 105. Data were plotted in GraphPad Prism 10.

### Cell toxicity with suspension cells

Cells were plated in an opaque 96-well plate at a density of 20,000 cells/well (50 μL per well of a 400,000 cells/mL stock). In all, 200 μL of PBS was added to the surrounding wells. Cells were then treated with 50 μL of a 2× target concentration of compound stocks prepared at an initial 2% DMSO solution in cell media (at 1% FBS), in triplicate. This made each sample a total of 100 μL, 1% DMSO final, at 1× the target concentration of compound. The cell plate was placed into the cell incubator for the indicated hours of incubation. After the timepoint, the cell plate was removed from the cell incubator and brought to RT for 30 min. Overall, 90 μL of Cell Titer-Glo luminescent reagent was added to each well. The plate was gently shaken for 2 min. The plate was then protected from light for 10 min prior to being measured. Luminescence was recorded using a BioTek Synergy Neo2Multimode Microplate Reader with a gain of 105. Data were plotted in GraphPad Prism 10.

### Cell toxicity pre-dosed with ONX-0914

Cells were plated in a T25 flask at 800,000 cells/mL. To the flask, ONX-0914 was added at a final concentration of 10 µM, 1% DMSO, and allowed to incubate for 1 h at 37 °C with 5% CO_2_. After the hour incubation, cells were harvested, spun down at 800×*g*, and the media removed. Cells resuspended at 400,000 cells/mL. Cells were then plated in an opaque 96-well plate at a density of 20,000 cells/well (50 μL per well of a 400,000 cells/mL stock). In total, 200 μL of PBS was added to the surrounding wells. Cells were then treated with 50 μL of a 2× target concentration of compound stocks prepared at an initial 2% DMSO solution in cell media (at 1% FBS), in triplicate. This made each sample a total of 100 μL, 1% DMSO final, at 1× the target concentration of compound. The cell plate was placed into the cell incubator for 24-h incubation. After the timepoint, the cell plate was removed from the cell incubator and brought to RT for 30 min. In all, 90 μL of Cell Titer-Glo luminescent reagent was added to each well. The plate was gently shaken for 2 min. The plate was then protected from light for 10 min prior to being measured. Luminescence was recorded using a BioTek Synergy Neo2Multimode Microplate Reader with a gain of 105. Data were plotted in GraphPad Prism 10.

### Cellular assay with activity-based probe TBZ-1

Cells were plated in a black 96-well plate at a density of 5000 cells/well. Adherent cells were allowed to adhere overnight, while suspension cells were used the same day. The media was removed, and fresh media (50 µL) was added to each well. Each probe was added to each well at the corresponding concentration, and the plate was returned to the incubator for the given amount of time. To remove any signal due to FBS in the media, the media was removed, and cells were washed with PBS (3×). After the final wash, PBS (50 µL) was added to each well. The plate was incubated at 37 °C in the plate reader, monitoring the change in fluorescence (Ex – 485(20) nm, Em – 535(20) nm for Rh-probes; gain = 90). Data was plotted using GraphPad Prism 10 with reported values as an average of samples, with error bars shown as the standard deviation.

### Tubulin polymerization assay (tubulin tracker™ deep red)

NCI-H446 were plated at 8000 cells/well in an eight-well confocal compatible plate (Nunc™ Lab-Tek™ II Chambered Coverglass - 155409PK) with 500 µL of media. Cells were placed back in the incubator at 37 °C with 5% CO_2_ overnight. The next day, the media was removed, and compounds of MMAE or m-ATMW-PABC-MMAE at 25 nM in 500 µL of media were added to wells. DMSO was used as a control, ensuring all samples remained less than 2% DMSO overall. Cells were placed back in the incubator for 6 h. After 6 h, the media was removed, and tubulin tracker red dye was added as described below.

Initially, a 1000× stock solution of Tubulin Tracker™ Deep Red was made by dissolving the contents of the vial in 60 µL of anhydrous DMSO. This stock solution is stable for at least three months when stored at ≤–20 °C. From the 1000× stock solution, a 1× solution was prepared by diluting in live-cell compatible buffer (Enough volume to sufficiently cover cells). The samples were incubated for 30 min at 37 °C and 5% CO_2_. Hoechst also added at 1× concentration (or other nuclear stain of choice). Cells were rinsed three times with PBS before adding additional live-cell imaging solution and imaging by confocal microscopy.

### Propidium iodide/Annexin V staining

NCI-H446 cells were plated in a 12-well plate at 500,000 cells/well (1000 µL of a 500,000 cells/mL) stock. Cells were allowed to adhere overnight. The next day, compounds were prepared at 50 nM in 1000 µL of media and added to the wells. This was allowed to incubate for 24 h. After dosing, cells were removed from the flask and placed in 1.5-mL Eppendorf tubes at a concentration of 1 × 10^6^ cells/mL. Cells were washed with cold PBS 2× and then resuspended in 1× binding buffer at 1 × 10^6^ cells/mL. In all, 100 µL of solutions (1 × 10^5^ cells) were transferred to a 5-mL culture tube. In total, 5 µL of FITC Annexin V and 5 µL of propidium iodide were added to each sample, gently vortexed, and incubated for 15 min at RT in the dark. After 15 min, 400 µL of 1× binding buffer is added to each tube and analyzed by flow cytometry on a BD Accuri C6 flow cytometer. Samples analyzed on FlowJo V.10 and cellular distributions plotted in GraphPad Prism 10.

### Spheroid dosing

NCI-H446 cells were plated in low-adhesion, U-shaped-bottom 96-well clear plates (Nunclon™ Sphera™ 174925) at 5000 cells per well in 100 µL of media. Cells were placed in an incubator at 37 °C with 5% CO_2_ for 4 days. After 4 days, the spheroid had started to form as determined by imaging on CELLCYTE X™ live-cell imager. Compounds were prepared at 0, 0.01, 0.01, 1, and 10 nM in 100 µL of media. The media was removed from the wells gently so as not to disturb the cells and replaced with 100 µL of media. Cells were placed back in the incubator and imaged every other day for a total of 12 days. Fresh media was added on days 4 and 8 (50 µL) to ensure cell health was maintained. Volume of spheroids was determined by ImageJ. All concentrations were done in triplicate (*n* = 3).

### Growth assay

NCI-H446 cells were plated at 500,000 cells/well (1000 µL of a 500,000 cell/mL stock) in a 12-well clear plate. Cells were allowed to adhere overnight in an incubator at 37 °C with 5% CO_2_. The following day, compounds were prepared at 50 nM in 1000 µL of media. Media was removed and replaced with fresh media + compound. The plate was placed back in an incubator and imaged every day on a CELLCYTE X™ live-cell imager. The total area (mm^2^) was plotted in Graph Pad Prism 10 to show changes over time. All compounds were dosed in triplicate (*n* = 3).

### siRNA

NCI-H446 cells were plated in a 24-well plate at 200,000 cells/well (500 μL of a 400,000 cell/mL stock) and placed in an incubator to adhere overnight. The following day, cells were treated following DharmaFECT Transfection Reagent-siRNA Transfection Protocol. ON-TARGETplus siRNA (Cat ID:L-006022-00-0020) PSMB8 (LMP7) SMARTPool 20 nmol was purchased from Horizon and resuspended in 1× buffer (5× buffer: 300 mM KCl, 30 mM HEPES pH: 7.5, 1.0 mM MgCl_2_) to get to 20 μM stock. siRNA was prepared at a range of concentrations from 0 to 100 nM by diluting in serum-free media. 0.7 μL of the transfection reagent (DharmaFECT 1 Transfection Reagent, Cat ID: T-2001-01) was diluted in 174.3 μL of serum-free media and allowed to incubate for 5 min. siRNA in media was added to the transfection reagent and allowed to incubate for 20 min at RT. After 20 min, 1400 μL of media was added to get to a total volume of 1750 μL. Contents were added to cells in the 24-well plate and placed in an incubator for 48 h. Cells were collected and lysed with RIPA with 1× HALT. Cells were allowed to incubate with lysis buffer for 10 min before subjecting to centrifuge at 14,000×*g* for 15 min. Supernatant collected and protein normalized by BCA assay. In all, 30 ng of protein loaded into 10 well gels w/ 1× Laemmli loading buffer and BME. Gel ran at 175 V for ~40 min or until the bottom of the gel ran off. Gels were transferred using the Trans-Blot Turbo transfer method. Membranes were blocked with 50% blocking buffer and PBS for 1 h before incubating with Anti-LMP7, and Anti-Alpha Tubulin overnight at 4 °C. Primaries were removed and incubated with secondary (anti-goat and anti-mouse) antibodies for 1 h at RT. Membrane imaged on Sapphire 600 in the 800 and 680 channels. Bands were normalized to tubulin, and the amount of LMP7 was determined.

### siRNA with compounds

NCI-H446 cells were plated in a 24-well plate at 200,000 cells/well (500 μL of a 400,000 cells/mL stock) and placed in an incubator to adhere overnight. The following day, cells were treated following DharmaFECT Transfection Reagent-siRNA Transfection Protocol. ON-TARGETplus siRNA (Cat ID:L-006022-00-0020) PSMB8 (LMP7) SMARTPool 20 nmol was purchased from Horizon and resuspended in 1× buffer (5× buffer: 300 mM KCl, 30 mM HEPES pH: 7.5, 1.0 mM MgCl_2_) to get to 20 μM stock. siRNA was prepared at a concentration of 10 nM by diluting in serum-free media. In total, 0.7 μL of the transfection reagent (DharmaFECT 1 Transfection Reagent, Cat ID: T-2001-01) was diluted in 174.3 μL of serum-free media and allowed to incubate for 5 min. siRNA in media was added to the transfection reagent and allowed to incubate for 20 min at RT. After 20 min, 1400 μL of media was added to get to a total volume of 1750 μL. Contents were added to cells in the 24-well plate and placed in an incubator for 48 h.

After the time point, cells were removed from the flask and replated in a 96-well white plate (20,000 cells/well). 200 µL of PBS was added to the surrounding wells. Cells left to adhere overnight in the incubator. After adherence, media was removed, and cells were dosed with 100 µL 1× target concentration of compound stocks prepared at 0.5% DMSO solution in cell media (1% FBS) in triplicate. The cell was placed into an incubator for 24 or 96 hr time incubation. After the time point, 90 µL Cell Titer-Glo® luminescent reagent was added to each well. The plate was gently shaken for 2 min. The plate was then protected from light for 10 min prior to being measured. Luminescence was recorded using a BioTek Synergy™ Neo2 Multimode Microplate Reader with a gain of 105. Data were plotted in GraphPad Prism 10.

### Human SCLC xenograft model-based drug treatment

Mouse SCLC-derived UCI-ScTR-1 cells or human SCLC NCI-H446 cells were either orthotopically (lung) or subcutaneously injected into immune-competent or NOD scid gamma (NSG) mice (The Jackson Laboratories), respectively. Tumor growth was monitored using bioluminescence imaging or caliper measurements. For pilot toxicity analysis, once tumors were established 2 weeks after orthotopic implantation, mice were randomized into three groups: vehicle, 0.4 mg/kg MMAE, or 0.83 mg/kg ATMW was administered via tail-vein injection on a Monday-Wednesday-Friday schedule until all MMAE mice succumbed due to treatment toxicity. For dose-dependent analysis, tumors grew to ~400 mm^3^ and mice were randomized into three groups: 0, 0.83, and 1.66 mg/kg ATMW. ATMW or vehicle control (0 mg/kg) was administered via retro-orbital intravenous injection on a Monday-Wednesday-Friday schedule for 12 days. Tumor volume and body weight were recorded throughout the treatment period. The University of California, Irvine Institutional Animal Care and Use Committee approved all animal procedures. Kaplan-Meier survival curves were generated using GraphPad Prism. The Mantel–Cox test was used for statistical analyses of the curves.

### H&E, necrosis, and apoptosis analysis

At the endpoint of the in vivo drug response study, tumors were fixed and paraffin-embedded for hematoxylin and eosin (H&E) staining at the Experimental Tissue Resource, UC Irvine. For active cleaved caspase-3 immunohistochemistry staining, sections were baked, deparaffinized, rehydrated, and subjected to antigen retrieval in sodium citrate buffer. Endogenous peroxidase activity was quenched with 3% H_2_O_2_, followed by blocking with 5% goat serum/TBS-T for 1 h at room temperature. Sections were incubated overnight at 4 °C with a primary antibody against cleaved caspase-3 (1:200, BD Pharmingen, 559565), followed by an anti-rabbit biotinylated secondary antibody (1:200, Vector Laboratories, BA-1000) for 30 min at room temperature. Signal was developed using the VECTASTAIN ABC kit (Vector Laboratories) and DAB Quanto (Epredia), with hematoxylin counterstaining. Slides were mounted with Gelvatol and scanned using a Ventana DP200 scanner. Viable tumor regions were selected for caspase-3 analysis. All histological analyses were conducted using QuPath.

### LCMS tumor extraction

Excised tumor samples were weighed out (~ 100 mg) and kept on ice throughout extraction. Cold ethanol (400 µL) was added to the sample before homogenization with a Dremel. Dremel was used 10 s on, 30 s on ice to keep samples cold. Once samples were uniformly homogenized, they were spun down at 100×*g* for 5 min at 4 °C. The supernatant was transferred, and an additional 400 µL of cold ethanol was added to the homogenized sample. The protocol was performed again, and the supernatant was transferred and collected (total 800 µL). In all, 100 µL of sample was transferred to a new tube, and an additional 100 µL of water w/0.1% FA was added. 100 µL of sample transferred to a LCMS vial and 5 µL injected on an Xevo G2-XS QTOF (UCI Mass Spectrometry Facility). Analysis was performed using Mestre Nova to extract the mass of liberated MMAE or its metabolites.

### Tissue processing and generation of immune organoids

Tonsil tissues were processed, as previously described (LE Wagar^42^). Human tonsils were obtained from the Collaborative Human Tissue Network (CHTN) and transported in sterile saline on ice. Upon receipt, tissues were decontaminated for 30–60 min at 4 °C in Ham’s F12 medium (Gibco), penicillin–streptomycin (Gibco). Tonsils were rinsed in PBS and mechanically dissociated to generate single-cell suspensions. Suspensions were clarified by density gradient centrifugation using Lymphoprep (Stemcell Technologies) to remove debris. Cells were cryopreserved in fetal bovine serum (FBS) supplemented with 10% dimethyl sulfoxide (DMSO) and stored in liquid nitrogen until use. For immune organoid generation, cryopreserved cells were thawed rapidly, washed, counted, and seeded in ultra-low attachment plates (Corning) at 1.5 × 10^6^ cells/well in a final volume of 200 µL per well. Organoids were maintained in complete organoid medium consisting of RPMI-1640 with GlutaMAX (Gibco), 1× Antibiotic-Antimycotic (Gibco), and recombinant BAFF (0.5 µg/mL; Sino Biological). Cultures were incubated at 37 °C with 5% CO_2_ in a humidified incubator. Organoids were fed every other day by exchanging 30–50% of culture volume with fresh organoid media. Please see SI Appendix B for participant-level demographic information.

### Antigen stimulation

Immune organoids were stimulated with live attenuated influenza vaccine (LAIV; FluMist® Quadrivalent) to model inflammatory activation in culture. LAIV dosing was selected based on prior optimization to induce immune activation while preserving organoid viability. LAIV was added at the time of organoid setup at a final dilution of 1:1000. Parallel unstimulated cultures were prepared in the absence of LAIV. To assess compound activity in immune organoids, cultures were treated with either free monomethyl auristatin E (MMAE) or the corresponding prodrug. A dose–response screen was performed using tenfold serial dilutions ranging from 10 µM to 0.01 µM. Compounds were added once at the time of organoid setup under both LAIV-stimulated and unstimulated conditions. Based on this screening, 10 µM was selected as the working concentration for subsequent experiments. Cultures were maintained as described above and harvested on day 4 for flow cytometric analysis.

### Flow cytometry

Immune organoids were harvested at 4 days post stimulation and dissociated into single-cell suspensions. Cells were washed with FACS buffer consisting of phosphate-buffered saline (PBS) supplemented with 3% fetal bovine serum (FBS), 0.05% sodium azide, and 2 mM EDTA to remove residual culture components. Surface staining was performed using fluorophore-conjugated antibody panels. Viability dyes were included according to manufacturer instructions, where indicated, then fixed for 30 min on ice with 0.2% paraformaldehyde in PBS. Antibody cocktails were prepared in FACS buffer, and the cells were incubated with antibodies overnight at 4 °C, protected from light. Following staining, cells were washed with FACS buffer and resuspended for acquisition. Data were acquired on a Cytek Aurora spectral flow cytometer using SpectroFlow software. Spectral unmixing was performed using single-stain reference controls. Flow cytometry data were analyzed using FlowJo software.

### Informed consent and sample collection

Tonsils were obtained as de-identified discarded surgical specimens from individuals undergoing tonsillectomy for obstructive sleep apnea, hypertrophy, or recurrent tonsillitis. Because these specimens were fully de-identified prior to investigator access, their use was determined to be non-human subjects research by the University of California, Irvine Institutional Review Board (IRB; protocol 2020-6075 or non-human subjects determination).

## Supplementary information

SI

## Data Availability

All data supporting the conclusions of this study are presented in the main and supplementary figures.
