# Supplementary material for: From probe to prodrug: immunoproteasome activity as a trigger for disease-selective therapeutics
Source: Signal Transduct Target Ther. 2026 Aug 12;11:330. doi: 10.1038/s41392-026-02835-w (PMC13470457; doi:10.1038/s41392-026-02835-w)
Supplement: Supplementary file 1 [file 41392_2026_2835_MOESM1_ESM.docx]

Supplementary Materials for

From Probe to Prodrug: Immunoproteasome Activity as a Trigger for Disease-Selective Therapeutics

Cody A. Loy^1,5^, Yijun Gu^1^, Samuel C. Kim^6,7,8,9^, Mariam Mohagheghi^6,7,8,9^, Noah B. Trask^6,7,8,9^, Marina Suarez Pizarro^2,3^, Lisa E. Wagar^6,7,8,9^, Claudia A. Benavente^1,2,3^, Darci J. Trader^1,3,4,5*^

Correspondence to: dtrader@uci.edu

**This PDF file includes:**

Figures. S1 to S17

**Other Supplementary Materials for this manuscript include the following:**

Supplementary Schemes 1 & 2

Appendix A: Chemical Structure Characterization

Appendix B: Patient Sample Data

Figure. S1.

Western Blot analysis of Ramos B-cell Lymphoma, A549 lung carcinoma, MRC-5 lung fibroblast, and HEK-293T Kidney cells. Cells blotted for immunoproteasome subunit β5i, standard proteasome subunit β5, and tubulin as a loading control. All cell lines dosed with increasing concentrations of IFN-γ (0-20 ng/mL) for 72 hours before lysing and subjecting to western blot.

Figure. S2.

Structure of β5 (blue) vs β5i (purple) to show alterations in sequence as well as confirmational changes in substrate pockets that allow for different cleavage preferences. PDB: (6RGQ)- sCP, (6E5B)-iCP.

Figure. S3.

**a)** LCMS trace of control prodrug (ATMA) incubated with immunoproteasome after 1 hr. Total ion chromatogram (grey), Intact prodrug (green), and free MMAE (orange) could be detected. **b)** Microsomal stability of m-ATMW-PABC-MMAE as determined by LCMS. **c)** Human Serum Stability of m-ATMW-PABC-MMAE as determined by LCMS.

Figure. S4.

**a)** Cellular immunoproteasome activity analysis using TBZ-1 probe. Kinetic read every 90 sec for 90 mins in Ramos and HEK-293T cells to asses iCP activity. **b)** Cellular immunoproteasome activity analysis using TBZ-1 probe. Kinetic read every 90 sec for 90 mins in A549 cells with (+) or without (-) IFN-γ.

Figure. S5.

Cellular toxicity of A549 cells with (+) or without (-) preexposure to IFN-γ (5 ng/mL) for 72 hours then incubation with prodrug or MMAE for an additional 72 hours.

Figure. S6.

NCI-60 5 dose curves. 60 cancer cell lines treated at 5 different concentrations with iCP prodrug m-ATMW-PABC-MMAE.

Figure. S7.

mRNA expression levels of β5i across the available cancers from the NCI60. Data collected from The Human Protein Atlas and plotted as Normalized Transcript per Million (nTPM)

Figure. S8.

Apoptosis analysis by flow cytometry using Annexin V and Propidium Iodide staining in NCI-H446 after dosing with 25 nM of compound for 24 hrs.

Figure. S9.

Representative images of NCI-H446 spheroids from 1 nM dosing with MMAE, prodrug m-ATMW-PABC-MMAE, or control m-ATMA-PABC-MMAE over twelve days.

Figure. S10.

Representative images of NCI-H446 cellular growth from 1 nM dosing with MMAE, prodrug m-ATMW-PABC-MMAE, or control m-ATMA-PABC-MMAE over 7 days.

Figure. S11.

**a)** PSMB8 siRNA in NCI-H446 cells after incubation with 0-100 nM siRNA for 24 hours. **b)** Cellular toxicity of NCI-H446 with (+) or without (-) PSMB8 siRNA and treatment with iCP prodrug m-ATMW-PABC-MMAE or MMAE at 1 nM for 48 hours.

Figure. S12.

**a)** Relative luminescence signal of SCLC tumors after treatment with m-ATMW-PABC-MMAE (0.83 mg/kg) or Vehicle (0 mg/kg) in the pilot study of immunocompetent mice. **b)** Idexx toxicity assessment from serum of mice dosed with m-ATMW-PABC-MMAE (0.83 mg/kg) or Vehicle (0 mg/kg). Mean ± SD.

Figure. S13.

**a)** Relative luminescence signal of SCLC tumors after treatment with m-ATMW-PABC-MMAE (0.83 mg/kg) or Vehicle (0 mg/kg) in the pilot study of immunocompetent mice. **b)** Idexx toxicity assessment from serum of mice dosed with m-ATMW-PABC-MMAE (0.83 mg/kg) or Vehicle (0 mg/kg). Mean ± SD.

Figure. S14.

Total ion chromatogram (TIC, left) of tumor homogenate from control mice (top) and mice treated with the high-dose of m-ATMW-PABC-MMAE (1.66 mg/kg, bottom). Extracted ion chromatogram (EIC, middle) No liberated MMAE was detected in control samples, while it was detected in mice treated with the high dose (right). The M+Na ion for MMAE as well as a common metabolite was detected.

Figure. S15.

Idexx toxicity assessment from serum of mice dosed with m-ATMW-PABC-MMAE (0.83 mg/kg), m-ATMW-PABC-MMAE (1.66 mg/kg) or Vehicle (0 mg/kg).

Figure. S16.

Tonsil-Derived organoids from 11 independent patient samples. Cultures harvested four days post-treatment and analyzed by high-dimension spectral flow cytometry. Samples were treated with vehicle, MMAE, or Prodrug either Unstimulated (Unstim) or Stimulated (LAIV) with attenuated influenza A vaccine.

Figure. S17.

**a)** iCP activity assay with Ramos B-Cells and DC 2.4 Dendritic cells. **b)** Cellular toxicity analysis of m-ATMW-PABC-MMAE and MMAE in DC 2.4 Dendritic cells after 24 hrs.

**Supplementary Scheme 1.**

A) 1 (1 eq.), TCFH (1.2 eq.), NMI (3 eq.), Para-aminobenzyl alcohol (1.5 eq.) DCM, RT, overnight. **B)** 2 (1 eq.) Bis(4-nitrophenyl)carbonate (3 eq.) DIPEA (6 eq.), THF, RT, overnight. **C)** 3 (1 eq.), MMAE (2 eq.) DIPEA (4 eq.) Pyridine (2 eq.), DMF, RT, 48 hrs. **D)** 4 (1 eq.), TFA (95%), DCM (5%), RT, 1 hr.

**Supplementary Scheme 2.**

A) 6 (1 eq.), TCFH (1.2 eq.), NMI (3 eq.), Para-aminobenzyl alcohol (1.5 eq.) DCM, RT, overnight. **B)** 2 (1 eq.) Bis(4-nitrophenyl)carbonate (3 eq.) DIPEA (6 eq.), THF, RT, overnight. **C)** 3 (1 eq.), MMAE (2 eq.) DIPEA (4 eq.) Pyridine (2 eq.), DMF, RT, 48 hrs. **D)** 4 (1 eq.), TFA (95%), DCM (5%), RT, 1 hr.

**Appendix A: Chemical Structure Characterization**

**Chemical Structure Characterization 1.**

LCMS of m-AT(tBu)MW(Boc)-PABC (3)

**Chemical Structure Characterization 2.**

LCMS of m-ATMW-PABC-MMAE (5)

**Chemical Structure Characterization 3.**

LCMS of m-ATMA-PABC-MMAE (10)

**Chemical Structure Characterization 4.**

LCMS of Ac-MMAE

**Appendix B: Patient Sample Data**

| **Donor** | **Age** | **Sex** |
| --- | --- | --- |
| WD-120652 | 7 | F |
| WD-120651 | 13 | F |
| WD-120478 | 12 | F |
| WD-120645 | 9 | F |
| WD-120477 | 12 | F |
| WD-110556 | 3 | M |
| WD-119715 | 11 | M |
| WD-118256 | 5 | F |
| WD-118275 | 7 | F |
| WD-118577 | 12 | M |
| WD-120303 | 9 | M |
